# Characterization of Somatic Embryogenesis Receptor-Like Kinase 4 as a Negative Regulator of Leaf Senescence in *Arabidopsis*

**DOI:** 10.3390/cells8010050

**Published:** 2019-01-14

**Authors:** Xiaoxu Li, Salman Ahmad, Akhtar Ali, Cun Guo, Hong Li, Jing Yu, Yan Zhang, Xiaoming Gao, Yongfeng Guo

**Affiliations:** 1 Key Laboratory for Tobacco Gene Resources, Tobacco Research Institute, Chinese Academy of Agricultural Sciences, Qingdao 266101, China; 82101171073@caas.cn (X.L.); safitfa@yahoo.com (S.A.); akhtar_arid@yahoo.com (A.A.); 82101172197@caas.cn (C.G.); lihongcq1993@163.com (H.L.); yujing_qau@163.com (J.Y.); zhangyan03@caas.cn (Y.Z.); gaoxiaoming@caas.cn (X.G.); 2 Graduate School of Chinese Academy of Agricultural Sciences, Beijing 100081, China

**Keywords:** SERK4, leaf senescence, LRR-RLK, cell-to-cell communication, cell death

## Abstract

Leaf senescence is a genetically controlled process that involves the perception of extracellular signals and signal transduction. The receptor-like protein kinases (RLKs) are known to act as an important class of cell surface receptors and are involved in multiple biological processes such as development and stress responses. The functions of a number of RLK members have been characterized in *Arabidopsis* and other plant species, but only a limited number of RLK proteins have been reported to be associated with leaf senescence. In the present study, we have characterized the role of the somatic embryogenesis receptor kinase 4 (*SERK4*) gene in leaf senescence. The expression of *SERK4* was up-regulated during leaf senescence and by several abiotic stress treatments in *Arabidopsis*. The *serk4-1* knockout mutant was found to display a significant early leaf senescence phenotype. Furthermore, the results of overexpression analysis and complementary analysis supported the idea that SERK4 acts as a negative regulator in the process of leaf senescence.

## 1. Introduction

Leaf senescence in plants is a predetermined cell-death mechanism and a coordinated process that involves the remobilization of nutrients [1]. Prior to death, plants undergo a process of recycling and translocating nutrients from senescing organs to non-senescing parts such as seeds and other storage tissues, ensuring their survival and propagation. The senescence process of leaves thus largely affects the yield and nutritional value of food crops [2,3]. Apparently, leaf senescence is marked by the yellowing of old leaves from tip to base due to the degeneration of chlorophylls. At the biochemical level, macromolecules such as lipids, proteins and carbohydrates are degraded [4], while at the molecular level, a number of signaling pathways operate with the involvement of key regulatory components to activate the expression of a subset of senescence associated genes (*SAGs*), participating in senescence regulation [5,6].

Among the regulatory components, the cell surface receptors are important structural proteins, serving as the scaffold to perceive signals which coordinate cell-to-cell communication. The receptor-like kinases (RLKs) are the most characterized class of cell-surface receptors, with unique structural features [7,8]. A typical RLK comprises an extracellular binding domain (ECD) to perceive specific ligands, a transmembrane domain (TM) to fix the protein on the plasma membrane and a cytoplasmic kinase domain (KD) to transduce the signals into the cell to activate the downstream regulatory components via phosphorylation [7]. The phylogenetic study of the RLKs indicated that nearly 610 members are encoded by the *Arabidopsis* genome, representing a large monophyletic gene super family [9]. Among the 44 subclasses of RLKs, leucine-rich repeat receptor-like protein kinase (LRR-RLK) represents the largest subfamily in the *Arabidopsis* with over 200 members [9]. The ECD of LRR-RLKs are furnished with individual leucine-repeat units, 24 amino acids long, varying in both number and arrangement [7]. To date, a large number of LRR-RLK members have been identified in *Arabidopsis*, rice, poplar, potato, tomato and other plant species [9,10,11,12]. However, only limited members have been assigned to various biological functions including plant growth and development and stress responses [7].

Being a development and age-dependent process, leaf senescence can be triggered by various intrinsic and extrinsic factors including age and hormones as well as biotic and abiotic stresses [13]. The initiation and execution of leaf senescence are driven by the differential expression of genes [14]. Among them, a number of transcription factors, including members of the NAC, WRKY and MYB families, have been found to play important roles in regulating leaf senescence [15,16,17,18,19]. The ability of LRR-RLKs to sense various signals at the cell surface and regulate the activities of transcription factor through signal transduction makes them potential candidates to be components of the senescence regulatory network. In fact, several members of the LRR-RLK family have been identified as playing a role in the progress of leaf senescence. One LRR-RLK member, senescence-associated receptor like kinase (SARK), was reported to regulate leaf senescence in *Phaseolus vulgaris* [20]. Further, soybean (*Glycine max*) senescence-associated receptor-like kinase (GmSARK) and its homologue AtSARK in *Arabidopsis* were functionally characterized, and their knockout mutant exhibited precocious leaf senescence [21,22]. Another LRR-RLK member RPK1 was up-regulated by age and ABA, while acting as a positive regulator of leaf senescence in *Arabidopsis* [23].

In an attempt to isolate senescence-regulating LRR-RLKs, *Arabidopsis* T-DNA insertion lines of selected LRR-RLK family genes were screened for leaf senescence-related phenotypes followed by the systematic analysis of related genes. Here, we describe the characterization of an important LRR-RLK member which has been reported as the somatic embryogenesis receptor-like kinase 4 (SERK4) and grouped into the SERK subfamily in *Arabidopsis* [24]. In prior studies, SERK4 was found to negatively regulate cell death through elusive mechanisms [25,26,27]. Notably, the SERK subfamily members have been reported in various studies with different functions including brassinosteroid signaling, stomatal patterning, immunity and cell death [25,28,29]. In this study, the *SERK4* gene was found to be highly expressed in senescent leaves and to control leaf senescence in *Arabidopsis*.

## 2. Materials and Methods

### 2.1. Plant Materials

The *Arabidopsis* wild type Col-0 was kept in our laboratory and the T-DNA insertion line SALK_057955, which has been reported previously [29], was obtained from the Arabidopsis Biological Resource Center (ABRC). Homozygous mutant plants were identified with the PCR based method using three primers including the T-DNA left border primer (5′-ATTTTGCCGATTTCGGAAC-3′), the gene specific left primer (5′-TGGCTCAGAAGAAAACCACAG-3′), and the right border primer (5′-CTGCTCCACTTCTGTTTCCAC-3′).

### 2.2. Plant Growth and Stress Treatments

*Arabidopsis* seeds were surface-sterilized by immersion in 70% ethanol and were well dried before sowing on Petri dishes containing half-strength MS (Murashige and Skoog) solid media. Two-week old seedlings with four leaves from the plates were transplanted to pots containing soil mixture (pindstrup–peat moss and vermiculite, 3:1 *v*/*v*). *Arabidopsis* plants were kept in a growth room at 22 °C with continuous light. Different tissues, including the shoot, root, flower, young leaf, and senescence leaf of four-week old *Arabidopsis* plants, were harvested for gene expression analysis. Leaves at different developmental stages were used for gene expression analysis. This includes young leaves that are still undergoing expansion, mature leaves that are fully expanded with no senescence phenotype, early senescence leaves with senescence initiated at the tip, and late senescence leaves in which senescence has proceeded to the middle section of a leaf. Environmental stresses such as extreme temperature, salt, and drought can induce senescence. According to previous studies, *Arabidopsis* seedlings were treated with 150 mM NaCl, 200 µM mannitol, 35 °C and 4 °C for 1, 3 and 6 h, respectively [13,30]. All of these samples were frozen in liquid nitrogen immediately after harvest and stored at −80 °C before analysis.

### 2.3. RNA Extraction and qRT-PCR

Total RNA was extracted using RNAiso (TaKaRa, Shiga, Japan) based on the manufacturer’s instructions. The first-strand cDNA synthesis was performed with 2 μg RNA using the PrimeScript™ RT reagent Kit (TaKaRa). Further, the qRT-PCR analysis was conducted using an ABI 7500 real-time PCR system (Applied Biosystems, Waltham, MA, USA) with a 20 μL reaction volume including SYBR (TaKaRa) 10 μL, 10 μM forward/reverse primer 0.4 μL, and 100 ng/μL cDNA 0.2 μL. The *ACTIN2* (*ACT2*) gene was used as the internal control for normalization, whereas the *SAG12* and *RBCS* genes were used as senescence markers [15,31]. The relative fold change was calculated based on the 2^−ΔΔct^ method. The qRT-PCR assay results were collected from three independent biological repeats with three technical replications. The *t*-tests were performed with GraphPad Prism 5 (GraphPad Software Inc., San Diego, CA, USA). Sequences of primers are listed in Supplementary Table S1.

### 2.4. Subcellular Localization Analysis

The coding sequence (CDS) of *SERK4* was amplified and ligated into the pEasy-Blunt vector (Transgen Biotech, Beijing, China). Then, the CDS of *SERK4* excluding the stop codon together with a green fluorescent protein (GFP) fragment were inserted into the SacI site of the pCHF3 vector by Infusion (Clontech, Palo Alto, CA, USA). The 35S::SERK4-GFP construct and a previously reported 35S::GFP control [32] were used for *Agrobacterium*-mediated transient expression in *Nicotiana benthamiana* leaves [33]. The GFP fluorescence signals were captured by a confocal microscope (TCS-SP8, Leica, Wetzlar, Germany) three to four days after the injections.

### 2.5. Overexpression and Complementation Test

For overexpression analysis, the CDS of *SERK4* was amplified and inserted into the SacI site of the pCHF3 vector by Infusion (Clontech). For complementation testing, the native promoter (2.2 kb) of *SERK4* was PCR-amplified and cloned into the EcoRI and SacI sites of the pPZP211 vector by Infusion (Clontech), resulting in the recombinant construction named pPZP211-pSERK4. Further, the CDS of *SERK4* was amplified and inserted into the SalI site of pPZP211-pSERK4 by Infusion (Clontech). After the sequencing of these recombinant constructions, the respective plasmids were transferred into GV3101 *Agrobacterium* competent cells. The positive colonies of *Agrobacterium* were selected and used to transform Col-0 or *serk4-1* mutant plants through the floral dip method [34]. The transformed *Arabidopsis* were selected on plates containing 50 mg/L kanamycin.

### 2.6. Measurements of Chlorophyll Content, Fluorescence and Ion Leakage

The chlorophyll content was quantified as described previously [35]. The fluorescence in leaves was measured with a chlorophyll fluorometer (Opti-Sciences, Tyngsboro, MA, USA) according to the manufacturer’s instructions. For ion leakage measurement, leaves were immersed in deionized distilled water, shaken at 25 °C for 30 min, and the beginning conductivity was measured using a digital conductivity meter (Thermo Fisher Scientific Traceable, Hampton, NH, USA). The samples were then boiled for 15 min and then the second conductivity was measured. The percentage of the first measurement over the second measurement was used as the membrane leakage indicator [35]. These assay results were retrieved from five independent biological repeats. The *t*-tests were performed with GraphPad Prism 5 (GraphPad Software Inc.).

## 3. Results

### 3.1. The SERK4 Gene is Up-Regulated During Leaf Senescence

**Figure 1:**
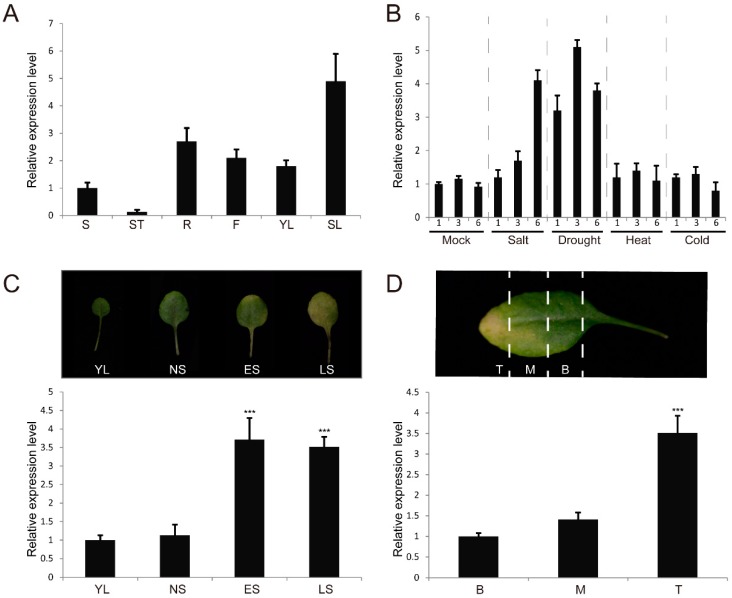
Expression analysis of the *SERK4* gene: (**A**) *SERK4* expression levels in tested tissues. The ratios of *SERK4* gene expression level in different tissues were calculated relative to the shoot. S, shoot; St. shoot tip; R, root; F, flower; YL, young leaf; SL, senescence leaf; (**B**) *SERK4* gene expression changes under abiotic stress treatments. The ratios of *SERK4* gene expression level under tested treatments were calculated relative to the untreated seedlings; (**C**) *SERK4* gene expression in leaves at different developmental stages. The ratios of *SERK4* gene expression level were calculated relative to the YL stage. YL, young leaf; NS, fully-expanded, non-senescent leaf; ES, early senescent leaf; LS, late senescent leaf; (**D**) *SERK4* gene expression at different sections of a senescing leaf. The ratios of *SERK4* gene expression level were calculated relative to the base section. B, base; M, middle; T, tip. In A, B, C and D, the expression data are means ± SD of three biological repeats. *** *p* < 0.001 (*t*-tests).

The expression pattern of *SERK4* was examined by qRT-PCR. The results showed that *SERK4* exhibited low expression in stem, root, flower and young leaf, but high expression in senescence leaf (Figure 1A). Besides this, the expression levels of *SERK4* under abiotic stress treatments were also determined by qRT-PCR. It was found that the transcript levels of *SERK4* were significantly induced by drought and salt stresses and reached peak levels with five-fold and four-fold increase after 3 h and 6 h treatments, respectively. However, *SERK4* expression seemed not to be responsive to heat or chilling stress treatments (Figure 1B).

Further, the *SERK4* expression pattern in a single leaf was examined under four growth stages, including the expanding young leaf stage, the fully expanded mature leaf stage, the early senescence leaf stage and late senescence leaf stage. The *SERK4* transcripts were detected to be highly expressed in leaves at both early and late senescence stages (Figure 1C). Since natural senescence proceeds from the tip section toward the base section of the leaf in *Arabidopsis*, a representative wildtype *Arabidopsis* leaf at the early senescence stage was divided into three parts, and the *SERK4* gene was found to be highly expressed in the tip part of the leaf (Figure 1D). These results suggested that the expression of *SERK4* is up-regulated during the process of leaf senescence.

### 3.2. The SERK4 Protein is Localized on the Cell Membranes

**Figure 2:**
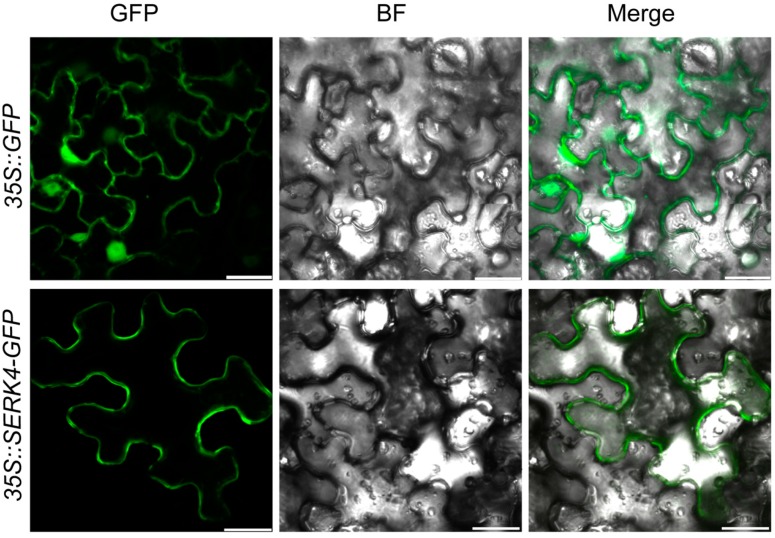
Subcellular localization analysis of the SERK4 protein: the green fluorescent protein (GFP) and SERK4-GFP fusion constructs under the control of the *CaMV-35S* promoter were transiently expressed in tobacco leaves. The scale bar represents 25 μm. GFP, GFP fluorescence; BF, bright field; Merge, the merged image of GFP and BF.

The *SERK4* gene is predicted to encode a typical LRR-RLK protein, which can be grouped with other SERK members to form a separated branch in the *Arabidopsis* LRR-RLK family. To determine the subcellular localization of the SERK4 protein, the CDS of *SERK4* gene excluding the stop codon was inserted to be in frame with a GFP reporter gene, under the control of the *CaMV-35S* promoter. The fluorescence of the fusion protein was specifically localized on the cell membranes, whereas the signals of 35S::GFP were found to be distributed in both membrane and cytosolic fractions (Figure 2).

### 3.3. Loss-of-Function of SERK4 Causes Early Senescence

**Figure 3:**
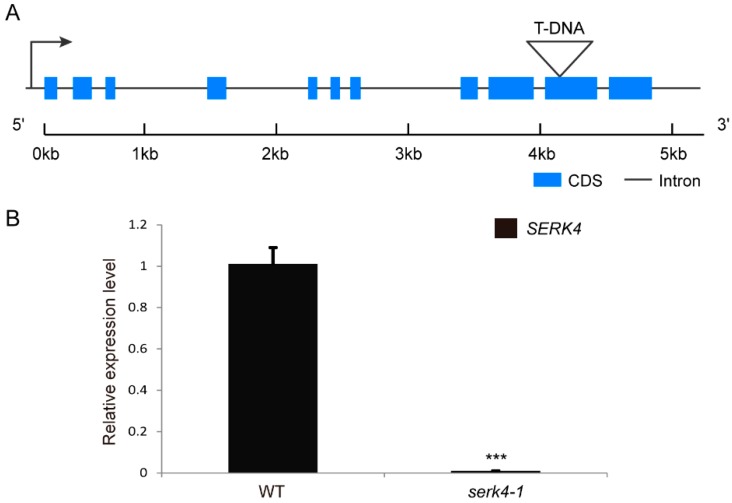
Expression of *SERK4* in the T-DNA insertion line: (**A**) the exon/intron structure of the *SERK4* gene and location of the T-DNA insertion; (**B**) the expression level of *SERK4* revealed by qRT-PCR in LS leaves of wildtype and the T-DNA insertion line, the ratios of *SERK4* gene expression level were calculated relative to the wildtype leaf. CDS, coding sequence; WT, wildtype. The expression data are means ± SD of three biological repeats. *** *p* < 0.001 (*t*-tests).

To investigate the role of *SERK4* in the regulation of leaf senescence, a mutant line named *serk4-1* was used for loss-of-function analysis. *serk4-1* (SALK_057955) was obtained from the *Arabidopsis* Biological Resource Center (ABRC) which harbored a T-DNA insertion in the second last exon of the *SERK4* gene. The homozygotes of *serk4-1* were confirmed by PCR-based genotyping and no *SERK4* transcript was detected in the LS-stage rosette leaves of the *serk4-1* mutant plants by qRT-PCR (Figure 3).

**Figure 4:**
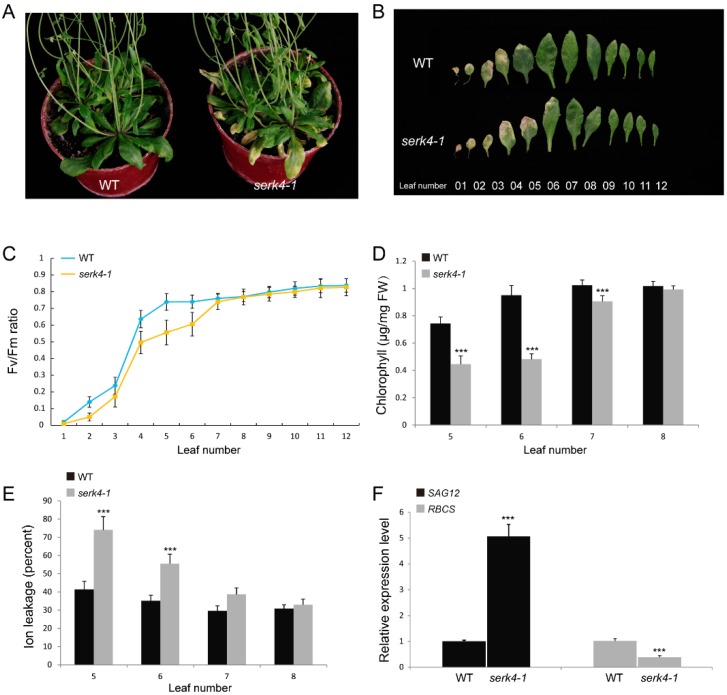
The early senescence phenotype of the *serk4-1* mutant; (**A**) the leaf senescence phenotype of the five-week-old *serk4-1* mutant and the wild-type plants; (**B**) phenotype of 12 detached rosette leaves from the *serk4-1* mutant and the wild type; (**C**) The Fv/Fm of the *serk4-1* mutant and the wild type; (**D**) the chlorophyll content of the *serk4-1* mutant and the wild type; (**E**) the ion leakage of the *serk4-1* mutant and the wild type; (**F**) the expression levels of *SAG12* and *RBCS* in the number 6 rosette leaf of the *serk4-1* mutant and the wild type. The ratios of *SAG12* and *RBCS* gene expression level were calculated relative to the wild type, respectively. WT, wildtype. In D, E and F, the data are means ± SD of three biological repeats. *** *p* < 0.001 (*t*-tests).

No significant difference of phenotypes between *serk4-1* and wild-type plants was observed at the early developmental stages, and bolting time was not affected in the *serk4-1* mutant. The five-week-old *serk4-1* mutant plants, however, showed an early senescence phenotype compared with the wild-type plants after bolting (Figure 4A). Further, 12 detached rosette leaves from mutant and wild-type plants were prepared as described previously and compared for their senescence phenotypes [15]. The results also showed a clear early senescence phenotype of the *serk4-1* mutant (Figure 4B). The Fv/Fm ratio is an indicator of the photosynthesis efficiency. A lower Fv/Fm ratio was found at four leaf positions of the mutant in comparison to the wild-type plants (Figure 4C). Consistent with the early senescence phenotype of the *serk4-1* mutant, the chlorophyll content of *serk4-1* plants was lower than that of the wildtype at leaf position 5–8 (Figure 4D). Furthermore, ion leakage, which is an important plasma membrane integrity indicator, was found to be lower at the four leaf positions of *serk4-1* than the wild-type plant (Figure 4E).

Molecular makers could help in estimating the senescence process prior to the visible phenotypes. The *SAG12* gene was reported to encode a cysteine proteinase and has been well accepted as a molecular marker for age-induced leaf senescence [30]. On the other hand, the *RBCS* gene was reported to be involved in photosynthesis and the expression level was found to be negatively correlated with the leaf senescence process [31]. As expected, the *SAG12* expression level of the *serk4-1* mutant was higher than wild-type plants, while the *RBCS* expression level of *serk4-1* plants was lower than for the wild type (Figure 4F).

### 3.4. Leaf Senescence is Delayed in Plants Overexpressing SERK4

**Figure 5:**
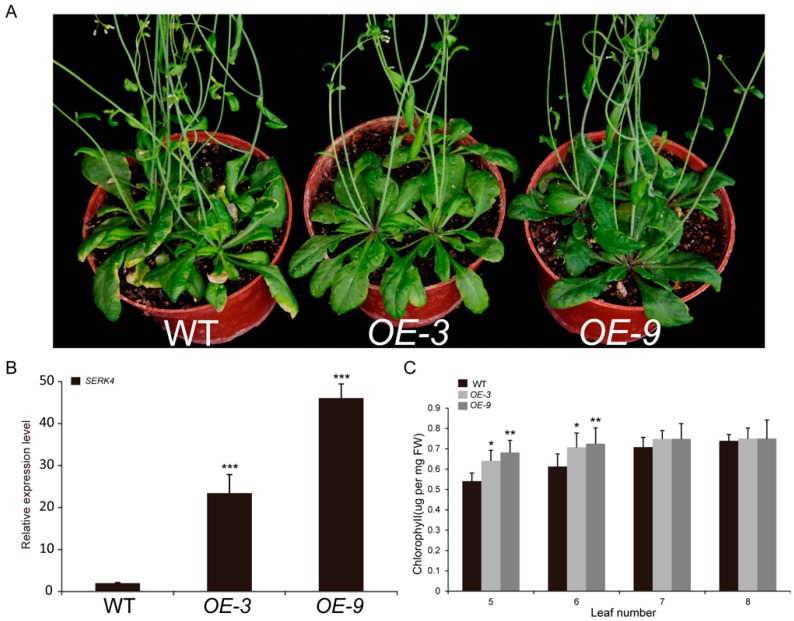
The delayed senescence phenotype of the *SERK4* overexpression lines; (**A**) the leaf senescence phenotype of the six-week-old wildtype and two *SERK4* overexpression lines plants; (**B**) the expression level of *SERK4* gene in wildtype and two *SERK4* overexpression lines, the ratios of *SERK4* gene expression level were calculated relative to the wildtype; (**C**) the chlorophyll contents of the wildtype and two *SERK4* overexpression lines. WT, wildtype; OE, overexpression line. In B and C, the data are means ± SD of three biological repeats. * *p* < 0.05, ** *p* < 0.01, *** *p* < 0.001 (*t*-tests).

To further investigate the roles of *SERK4* in leaf senescence, gain-of-function analysis was carried out. The CDS of *SERK4* was fused into the pCHF3 vector after the 35S promoter and transgenic lines were generated to overexpress *SERK4* in the wild-type background. Hence, two overexpression lines, named *OE-3* and *OE-9*, were selected for phenotyping. Compared to the six-week-old wildtype plants, the overexpression of *SERK4* in transgenic *Arabidopsis* resulted in a significant delay of leaf senescence (Figure 5A,B). Notably, the bolting time was not affected in these two *SERK4* overexpression lines. The overexpression lines showed higher chlorophyll content than the wild-type plants (Figure 5C).

### 3.5. The *SERK4* Gene Rescues the Serk4 Mutant Phenotypes

**Figure 6:**
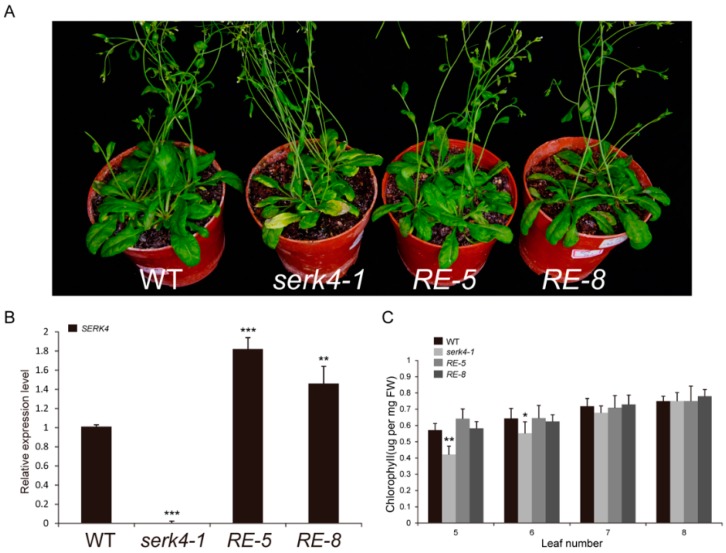
Complementation assay of the *serk4-1* mutant with the *SERK4* gene; (**A**) the leaf senescence phenotype of the five-week old wild-type, *serk4-1* mutant and two rescue-line plants; (**B**) the expression level of the *SERK4* gene in the wild type, *serk4-1* mutant and two rescue lines, the ratios of *SERK4* gene expression level were calculated relative to the wild type; (**C**) the chlorophyll contents of the wild type, *serk4-1* mutant and two rescue lines. WT, wildtype; RE, rescue line. In B and C, the data are means ± SD of three biological repeats. * *p* < 0.05, ** *p* < 0.01, *** *p* < 0.001 (*t*-tests).

To further confirm these senescence-related phenotypes were conferred by alteration of the *SERK4* gene expression, a complementation test was carried out. Driven by the 2.2 kb native *SERK4* promoter, the CDS of the *SERK4* gene was inserted into the pPZP211 vector, and the resulting complementation construct was used to transform the *serk4-1* mutant plants. The early senescence phenotype of *serk4-1* was restored to the wild type in two independent transgenic lines (Figure 6A,B). The senescence phenotypes were further supported by the chlorophyll content results (Figure 6C).

## 4. Discussion

Leaf senescence is a genetically controlled cell-death process. During the past two decades, a large number of *SAG*s have been identified and a subset of *SAG*s have been characterized to be involved in the regulation of the initiation and execution of leaf senescence [13]. Here, we characterized one of the *Arabidopsis* LRR-RLK family members, SERK4, which participated in the regulation network of leaf senescence. The expression of *SERK4* was found to be up-regulated in the senescence leaf. Compared to the wild-type plant, the *serk4-1* mutant displayed an early senescence phenotype with a higher expression level of the senescence marker gene *SAG12*, lower chlorophyll content and Fv/Fm ratio, and high ion leakage. Although only one single loss-of-function mutant was analyzed, the fact that the early senescence phenotype of the *serk4-1* mutant was successfully rescued to the wild type by the *SERK4* gene, and that two transgenic lines overexpressing *SERK4* exhibited a delayed leaf senescence phenotype, fully supported the role of SERK4 as a negative regulator of leaf senescence in *Arabidopsis*.

Comprising five members (SERK1-5) in *Arabidopsis*, the SERK family has been characterized as co-receptors in a number of signaling pathways [36,37]. In previous studies, SERK1 was reported as a co-receptor and found to interact with BRI1, PSKR and HAESA respectively, resulting in heterodimeric complexes which work in various signaling pathways [37,38,39]. Besides this, the SERK family member SERK3/BAK1 was observed to interact with BRI1 in regulating BR signaling, while BAK1 was also found to interact with FLS2 and contribute to conferring plants with innate immunity [40,41]. Furthermore, SERK4 was reported to interact with SERK3/BAK1, while the silencing of *SERK3/BAK1* and *SERK4* at the same time caused cell death and H_2_O_2_ production in *Arabidopsis*, suggesting that SERK4 is also a negative regulator of cell death [25,26,27]. Programmed cell death is a genetically controlled process of cell suicide, while leaf senescence as the last stage of leaf development is a type of programmed cell death which is featured by catabolic events such as proteins and nucleic acid degradation [13,25,42]. Based on the present clues, it is suggested that SERK4 acts as a negative regulator in both cell death and leaf senescence, and may coordinate these two important processes in *Arabidopsis*. Besides this, SERK4 may also act as a co-receptor in negatively regulating leaf senescence, which could be explored in further studies.

## Supplementary Materials

The following are available online at http://www.mdpi.com/2073-4409/8/1/50/s1, Supplementary Table S1: The PCR primers used in this study.
